# A Descending Circuit Derived From the Superior Colliculus Modulates Vibrissal Movements

**DOI:** 10.3389/fncir.2018.00100

**Published:** 2018-11-22

**Authors:** Miki Kaneshige, Ken-ichi Shibata, Jun Matsubayashi, Akira Mitani, Takahiro Furuta

**Affiliations:** ^1^Department of Morphological Brain Science, Graduate School of Medicine, Kyoto University, Kyoto, Japan; ^2^Laboratory of Physiology, Department of Human Health Sciences, Graduate School of Medicine, Kyoto University, Kyoto, Japan; ^3^Department of Oral Anatomy and Neurobiology, Graduate School of Dentistry, Osaka University, Suita, Japan

**Keywords:** whisker, premotor neurons, rat, anterograde tracing, kinematic analysis, CPGs

## Abstract

The superior colliculus (SC) is an essential structure for the control of eye movements. In rodents, the SC is also considered to play an important role in whisking behavior, in which animals actively move their vibrissae (mechanosensors) to gather tactile information about the space around them during exploration. We investigated how the SC contributes to vibrissal movement control. We found that when the SC was unilaterally lesioned, the resting position of the vibrissae shifted backward on the side contralateral to the lesion. The unilateral SC lesion also induced an increase in the whisking amplitude on the contralateral side. To explore the anatomical basis for SC involvement in vibrissal movement control, we then quantitatively evaluated axonal projections from the SC to the brainstem using neuronal labeling with a virus vector. Neurons of the SC mainly sent axons to the contralateral side in the lower brainstem. We found that the facial nucleus received input directly from the SC, and that the descending projections from the SC also reached the intermediate reticular formation and pre-Bötzinger complex, which are both considered to contain neural oscillators generating rhythmic movements of the vibrissae. Together, these results indicate the existence of a neural circuit in which the SC modulates vibrissal movements mainly on the contralateral side, via direct connections to motoneurons, and via indirect connections including the central pattern generators.

## Introduction

The superior colliculus (SC), in the midbrain, has long been considered an essential structure for eye movement control (for reviews, see Fuchs et al., 1985; Sparks and Mays, 1990; Sommer and Wurtz, 2008). Eye movements are necessary for visual sensation because the majority of ganglion cells are of the transient type, which quickly adapt, and thus static images on the retina quickly disappear from perception. The small visual field of the fovea is another reason why animals have to move their eyes (for review, see Gegenfurtner, 2016). Many brain regions related to the oculomotor system receive input from the SC, which obtains visual information from the retina and visual cortices (for review, see May, 2006).

Like visual sensation, tactile sensation of the vibrissal system in rodents is generated by actively moving the sensors themselves. Facial vibrissae (whiskers) on rodents are excellent tactile apparatus. Rats and mice sweep their vibrissae backward and forward to touch objects with the vibrissae when exploring. This vibrissal movement pattern is called whisking (Zucker and Welker, 1969). Depending on the situation, animals modulate the amplitude, velocity, and midpoint of their whisking (Mitchinson et al., 2007; Grant et al., 2009; Hill et al., 2011).

The SC is part of the neural circuit that processes vibrissal sensorimotor information. Previous studies have shown that SC neurons are responsive to a passive touch of the vibrissae (McHaffie et al., 1989; Grunwerg and Krauthamer, 1990; Castro-Alamancos and Favero, 2016), and to artificial vibrissal movements produced by facial nerve stimulation in anesthetized rats (Bezdudnaya and Castro-Alamancos, 2011, 2014). It has also been reported that electrical stimulation of the SC evokes vibrissal movements (McHaffie and Stein, 1982; Hemelt and Keller, 2008). Furthermore, the SC sends axons to the facial nucleus (FN), where the vibrissal motoneurons are located (Isokawa-Akesson and Komisaruk, 1987; Miyashita et al., 1994; Miyashita and Mori, 1995; Hattox et al., 2002). These earlier findings suggest that the SC may play an important role in control of vibrissal movements as well as eye movements.

In general, periodic movements of animals’ bodies are considered to be generated by the central pattern generators (CPGs), which are composed of small networks of neurons in the brainstem (for review, see Guertin, 2009). Whisking mainly consists of repetitive strokes of vibrissae along the rostrocaudal direction, and is controlled by the CPGs of whisking, which are located in the intermediate reticular formation (IRt) of the medulla oblongata and the pre-Bötzinger complex (preBötC) (Moore et al., 2013; Deschênes et al., 2016; for review, see Moore et al., 2014). The CPGs of whisking receive descending input from the vibrissal motor cortex (vMC), which modulates whisking behavior (Hattox et al., 2002; Grinevich et al., 2005). Hemelt and Keller (2008) previously reported that the vibrissal movements evoked by stimulating the vMC were substantially different from those induced by SC stimulation, suggesting that the role of the SC in whisking behavior differs from that of the vMC. Here, we hypothesized that the mechanism of the SC controlling vibrissal movements is closely correlated with architecture of neural circuits provided from the SC to the vibrissal motor related structures. To examine how the SC contributes to vibrissal movement control, we first analyzed differences in trajectories of vibrissal motion between normal rats and SC-lesioned rats. We also anterogradely traced the axonal projections from the SC to the CPGs and FN by using an axon-labeling technique with a virus vector.

## Materials and Methods

All animal use was in accordance with the National Institutes of Health Guide for the Care and Use of Laboratory Animals, and the experiments were approved by the Committee for Animal Care and Use (MedKyo 17039) and that for Recombinant DNA Study (141009) in Kyoto University. All efforts were made to minimize the number of animals used and the animal suffering.

### Surgical Procedures

Kinematic analyses of vibrissal movements were performed in 32 adult male Long-Evans rats (250–400 g, 16 control rats and 16 SC-lesioned rats). A light-weight, sliding head fixation frame (SR-8N; Narishige, Tokyo, Japan) was surgically attached to the skull with screws and dental resin cement (Miky plus; Nissin Dental Products Inc., Kyoto, Japan) under anesthesia produced by an intraperitoneal injection of chloral hydrate (35 mg/100 g body weight). At the same time, the unilateral side of the SC was lesioned by passing direct electrical current (2 mA, 5–10 s) two to three times through a stainless steel electrode. We lesioned the right side of the SC in eight rats, and the left side of the SC in another eight rats. The stereotaxic coordinates for the lesions were 6.5 mm behind the bregma, 1.5 mm lateral to the midline and 4 mm below the pia (Paxinos and Watson, 2007). All vibrissae on each side, except the C2, were cut off at the base. During the surgical recovery period, the rats had free access to water and food in their home cages.

### Apparatus and Experimental Design

**FIGURE 1:**
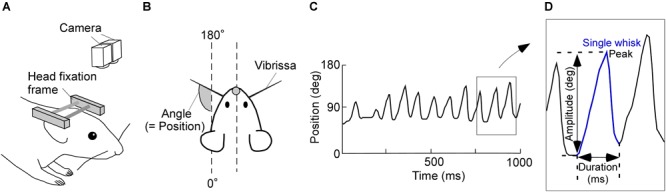
Experimental design and image tracking. **(A)** Schematic of the experimental setup for recording vibrissal movements in a head-restrained rat. Two high-speed cameras above the rat were used to monitor vibrissal movements. **(B)** Top view of the head-restrained rat. **(C)** Representative movement record. Upward and downward shifts in this plot represent forward and backward movements of a vibrissa, respectively. **(D)** Diagram of a typical single whisk, with the kinematic parameters labeled.

Data on vibrissal movement trajectories were obtained by recording the movements of a pair of bilaterally homologous vibrissae. Two high-speed video cameras (CV-H035M; Keyence, Osaka, Japan), set above the awake, head-restrained rat, monitored vibrissal motion with a 100-Hz frame rate (Figure 1A). One camera imaged the base of the C2 vibrissa on the right side, and the other imaged the base of the C2 vibrissa on the left. Vibrissal movements of the awake rats were recorded for 15 min (89170 sampling points) daily, for three consecutive days starting 3 days after the surgery. To acclimate the rats to head-fixation in the stereotaxic apparatus, for 2 days, starting on the day after surgery, they were head-fixed in the same manner that they would later be in the recording sessions. All the head fixed rats were not trained for any behavioral task and moved vibrissae freely as spontaneous behavior. The extent of each lesion was assessed on Nissl-stained frontal sections of the SC at the end of the experiment.

### Kinematic Analysis

Consecutive angle data were recorded for 15 min/day and imported into custom-written software (MATLAB; Mathworks, Natick, MA, United States). We defined the absolute angles of 0–180°, relative to the head-tail line of the rat, as the vibrissal position (see Figure 1B). Protractions on both the right and left sides of the face were represented as increasing vibrissal positions. Vibrissal tracking produced plots, like Figure 1C, of vibrissal positions against time (vibrissal motion on one side of the face is shown). We had a few error values (outliers), which were caused by failures in the measurement of the vibrissal angles. After removing the outliers, data were interpolated by spline or linear functions. We then applied a moving average of three sampling points.

Data segments of whisking periods and those of resting periods were extracted from the acquired vibrissal motion recordings. To evaluate the dynamic states of the vibrissae, we defined a single whisk as the combination of a forward vibrissal movement and a backward vibrissal movement (Figure 1D). A pair of forward and backward movements was identified as a whisk when the speed of vibrissal movement exceeded an angular velocity of 0.05 degrees/ms for longer than 20 ms. The peak and the duration of a movement, respectively, were defined as the maximum vibrissal position and the time between the beginning of the forward movement and the end of backward movement. The amplitude was defined as the angle between the beginning of the forward movement and the peak in a single whisk. Whisks were required to have a duration between 40 and 340 ms and an amplitude of ≥5°, to avoid the detection of vibrissal twitches (see Chen et al., 2016).

After detecting the whisks, resting periods were extracted from the residual recorded data by separated them into 500 ms epochs. We then found the maximum and minimum vibrissal positions in each epoch. When the difference between the maximum and minimum vibrissal positions was <5°, the epoch was defined as a resting period. Recorded data that did not correspond to either “whisking” or “resting” were categorized as “non-classified movement.”

### Statistical Analysis

The kinematic analyses were based on the 45-min record (15 min/day, for three successive days) from each animal. Data on vibrissal movements on the right and left sides of the face in control rats were merged (control: *n* = 32). Data from the two sides of the face in SC-lesioned rats were separated into vibrissal movements on the side ipsilateral to the lesion (ipsi: *n* = 16) and those contralateral to the lesion (contra: *n* = 16). All statistical analyses were performed in MATLAB. Differences between groups were tested with the Wilcoxon signed-rank test for paired data (ipsi versus contra) and the Mann–Whitney *U*-test for unpaired data (control rats versus SC-lesioned rats; control versus ipsi; control versus contra). The significance level was set at *p <* 0.05.

The vibrissal positions were evaluated after dividing the individual data into resting and whisking periods. The medians of the resting and whisking positions for each animal were computed and compared among the three (control, ipsi, and contra) groups in each period. We also calculated the distributions of whisk amplitudes for each animal, and compared the amplitude distributions between control rats and SC-lesioned rats (control versus ipsi; control versus contra). The distributions of whisk durations were computed in the same way, and compared between control rats and SC-lesioned rats. The coherence and phase lags between vibrissal movements on the right and left sides of the face were computed using Welch’s method [MATLAB magnitude-squared coherence (mscohere) and cross power spectral density (cpsd), respectively; 5.12 s Hamming window, 50% overlap, and 512 fast Fourier transform (fft) points]. Both the coherence and the phase lag per animal were first calculated using the 15 min of data from the separate days, and then the coherence and the phase lag for the 3 days were averaged and compared between SC-lesioned and control rats.

### Injection of Sindbis Virus and Fixation

Adult male Long-Evans rats (250–400 g) were used in the tract-tracing experiments. Eleven rats were anesthetized by intraperitoneal injection of chloral hydrate (35 mg/100 g body weight). For labeling of the SC neurons, we used Sindbis virus vector, which expresses palmitoylation site-attached green fluorescent protein (palGFP) (Furuta et al., 2001). PalGFP was targeted to the plasma membranes of the infected neurons (Moriyoshi et al., 1996), and was effective in visualizing the complete structure of neurons. We injected 0.2–0.4 μl of Sindbis virus vector (1–2 × 10^2^ infectious units) into the unilateral SC (5.8–7.0 mm posterior to the bregma, 0.7–2.0 mm lateral to the midline, and 3.5–4.3 mm deep from the brain surface) through a glass micropipette attached to a Picospritzer III (General Valve Corporation, East Hanover, NJ, United States). Sixty to sixty-five hours after virus vector injection, the rats were anesthetized with chloral hydrate (350 mg/kg body weight) and perfused transcardially with 200 ml of sodium phosphate (pH 7.4)-buffered 0.9% saline (PBS), followed by 300 ml of 3.7% (v/v) formaldehyde in 0.1 M sodium phosphate (PB), pH 7.0. The brains were removed, and post-fixed for 4 h at 4°C in the same fixative. After cryoprotection with 30% (w/v) sucrose in PBS, the blocks were cut into 40 μm-thick frontal sections on a freezing microtome, and the sections were collected serially in 0.1 M PB.

### Cell Count of palGFP-Expressing SC Neurons

The sections including the virus injection site were examined under an Axiophot epifluorescence microscope (Zeiss, Oberkochen, Germany) with a filter set for GFP (excitation, 450–490 nm; emission, 515–565 nm) to detect palGFP-labeled SC neurons. The number of infected neurons was manually counted in extracted sections, and the total number of infected neurons was properly estimated.

### Immunostaining for GFP

The sections including the axons of anterogradely labeled SC neurons were incubated overnight with 0.5 μg/ml affinity-purified rabbit antibody to GFP (Tamamaki et al., 2000; Nakamura et al., 2008) in PBS containing 0.3% (v/v) Triton X-100 (PBS-X). After three rinses with PBS-X, the sections were incubated for 2 h with 10 μg/ml biotinylated anti-[rabbit IgG] goat antibody (BA-1000; Vector Laboratories, Burlingame, CA, United States) and then for 2 h with avidin-biotinylated peroxidase complex (ABC) (1:100; ABC-Elite; Vector Laboratories) in PBS-X. After three rinses in PBS, we applied the biotinylated tyramine (BT)-glucose oxidase (GO) amplification method (Furuta et al., 2009; Kuramoto et al., 2009). The sections were incubated for 30 min in a BT-GO reaction mixture containing 1.25 μM BT, 3 μg/ml of GO (257 U/mg; 16831-14; Nacalai Tesque, Kyoto, Japan), 2 mg/ml of beta-D glucose, and 2% bovine serum albumin in 0.1 M PB, followed by three washes with PBS. This was followed by incubation for 2 h in ABC-Elite in PBS-X, and the bound peroxidase was visualized as a dark blue stain with a nickel-diaminobenzidine reaction. Finally, these sections were stained with neutral red.

### Quantitative Analysis of Anatomical Data

The axons of stained SC neurons were examined as described previously (Ohno et al., 2012). Briefly, a coronal section was automatically captured into a large color image, with a spatial resolution of 1.038 μm/pixel, by using a TOCO digital slide scanner (Claro, Aomori, Japan) equipped with a 10× objective lens (EC Plan-Neofluar; NA, 0.30; Zeiss). We traced the axon fibers on the images with CANVAS XII software (ACD Systems International Inc., VIC, Canada). The axon fibers were quantified using an “axon density index,” defined as follows. We first divided each section into 72 equal, quadrilateral areas, and calculated the length of traced axons in each of the areas with Adobe Illustrator CS4 software (Adobe Systems, San Jose, CA, United States). The axonal length in each area was divided by the number of infected SC neurons in each animal. We then normalized axon density index to eliminate the differences in the size of individual brains and brain sites in the lower brainstem.

To observe the axon fibers projecting to the IRt, preBötC, and FN, we first found the center of the facial nerve for each animal. Using this as a landmark, we picked out the three sections located 1.2, 2.16, and 2.64 mm posterior to the landmark. These extracted sections were located approximately 11.28 mm, 12.24 mm, and 12.72 mm posterior to the bregma according to the atlas by Paxinos and Watson (2007). These locations included the FN, IRt, and preBötC, respectively.

We also calculated the axon density index even in areas which contains the paramedian pontine reticular formation (PPRF) and abducens nucleus (AN), to compare the axon densities of SC projections to the vibrissal motor related structures with those of SC projections to the oculomotor related structures in the brainstem. Because the brain sections of the midbrain which contained the oculomotor nucleus were subjected to cell counting of infected neurons in the injection sites, axon density index of the oculomotor nucleus was not measured.

## Results

### Occurrence Rate of Vibrissal Reciprocating Motion (Whisks)

**FIGURE 2:**
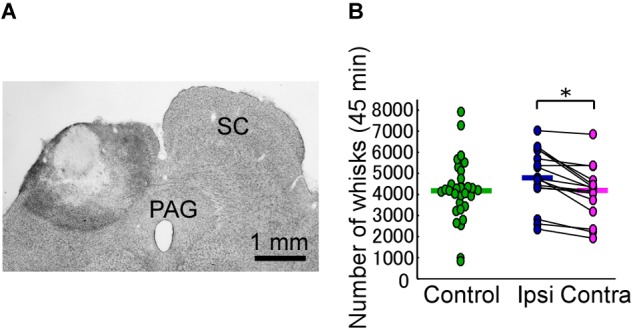
Unilateral lesion of the SC leads to differences in the occurrence rate of bilateral vibrissal reciprocating motions (whisks). **(A)** The extent of the SC lesion for a representative rat. **(B)** Numbers of whisks in each of the rats (45 min/day for 3 days). Horizontal lines indicate the median numbers of whisks. ^∗^*p* < 0.05 using Wilcoxon ranked-sum test (ipsi vs. contra). SC, superior colliculus; PAG, periaqueductal gray; ipsi, side ipsilateral to the lesion; contra, side contralateral to the lesion.

**FIGURE 3:**
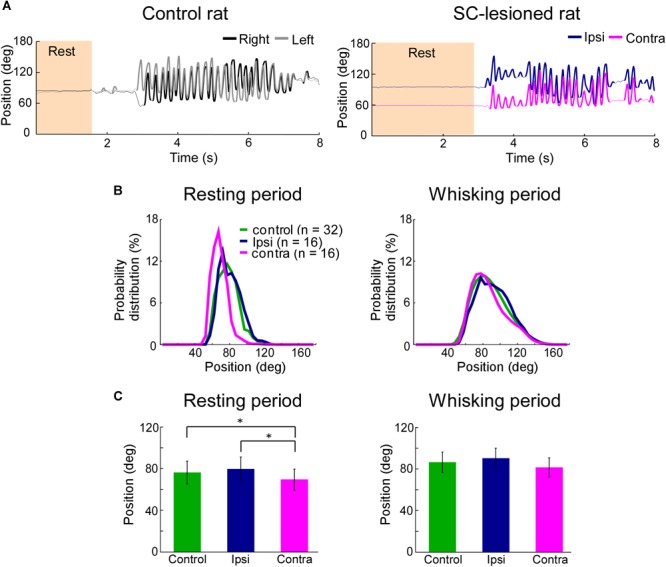
Backward shift of resting vibrissal position on the side contralateral to the lesion. **(A)** Movements of the right and left C2 vibrissae of a control rat (Left) and a SC-lesioned rat (Right). Examples of resting (shaded region) and whisking (bold trajectory) periods are shown. **(B)** The distributions of vibrissal positions in resting (Left) and whisking (Right) periods. The medians of the vibrissal position distributions of individual rats in each group (control, ipsi, or contra) are shown. **(C)** The medians of vibrissal positions of the individual rats were averaged in resting (Left) and whisking (Right) periods. All of the error bars indicate standard deviations. ^∗^*p* < 0.05 using Mann–Whitney *U*-test (control vs. ipsi; control vs. contra) and Wilcoxon ranked-sum test (ipsi vs. contra). SC, superior colliculus; ipsi, side ipsilateral to the lesion; contra, side contralateral to the lesion.

In each of the SC lesion experiments, we confirmed that both the medial and lateral parts of the unilateral SC were mostly (more than 80%) lesioned (Figure 2A). Unilateral lesioning of the SC did not clearly affect how often the vibrissal reciprocating movements known as whisks occurred (i.e., did not affect the “occurrence rate” of whisks) (Figure 2B). The numbers of whisks in control rats and SC-lesioned rats were not significantly different (control versus ipsi, Mann–Whitney *U*-test, *p* = 0.068; control versus contra, Mann–Whitney *U*-test, *p* = 0.84), whereas the number of whisks on the contralateral side in SC-lesioned rats was significantly lower than that on the ipsilateral side (Wilcoxon ranked-sum test, *p* = 0.00053). Figure 3A shows representative examples of vibrissal movement data in control and SC-lesioned rats. All of the rats exhibited resting periods and whisking periods.

### Vibrissal Position

To clarify the influence of SC lesions on the static parameters of vibrissae, we analyzed the median vibrissal positions in SC-lesioned rats and control rats. During resting periods, SC-lesioned rats exhibited significant differences in positions between the vibrissae ipsilateral to the lesion and those contralateral to the lesion (Figures 3B,C, Wilcoxon ranked-sum test, *p* = 0.0097). The contralateral vibrissae were also shifted backward relative to the vibrissal positions of the control rats (Mann–Whitney *U*-test, *p* = 0.0388). Conversely, during whisking periods, there were no significant differences in position between the ipsilateral and contralateral vibrissae or between the SC-lesioned rats and the control rats (control versus ipsi, Mann–Whitney *U*-test, *p* = 0.29; control versus contra, Mann–Whitney *U*-test, *p* = 0.10; ipsi versus contra, Wilcoxon ranked-sum test, *p* = 0.070).

### Whisking Amplitude and Whisking Frequency

**FIGURE 4:**
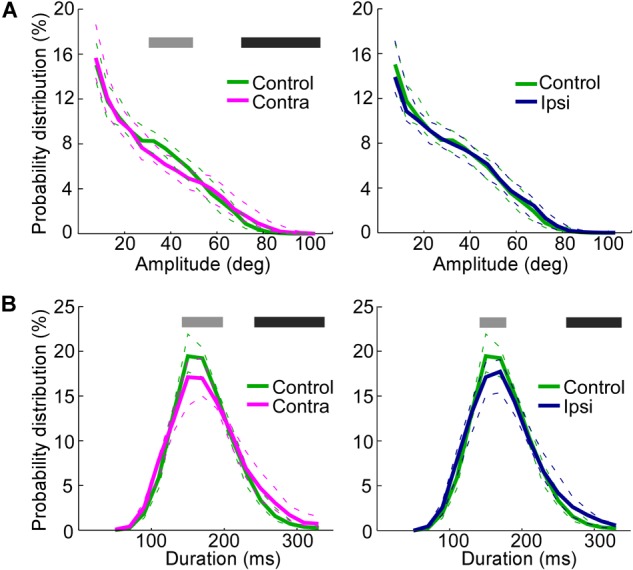
Increase in the whisking amplitude on the side contralateral to the lesion and decrease in the whisking frequencies on both the ipsilateral and contralateral sides. **(A)** Probability distributions of amplitudes, comparing the control rats with the contralateral side in SC-lesioned rats (Left) and the control rats with the ipsilateral side in SC-lesioned rats (Right). **(B)** Probability distributions of durations, comparing the control rats with the contralateral side in SC-lesioned rats (Left) and the control rats with the ipsilateral side in SC-lesioned rats (Right). Solid lines and dashed lines indicate medians and interquartile ranges, respectively. Black bars show *p* < 0.05 (control < ipsi; control < contra) using Mann–Whitney *U*-test. Gray bars show *p* < 0.05 (control > ipsi; control > contra) using Mann–Whitney *U*-test. SC, superior colliculus; ipsi, side ipsilateral to the lesion; contra, side contralateral to the lesion.

The amplitude distributions show that the vibrissae contralateral to the lesioned SC exhibited large-amplitude whisks (>70°) more frequently than did the vibrissae of the control rats (Figure 4A, Mann–Whitney *U*-test, *p* < 0.05), whereas moderate-amplitude whisks (>30°, <50°) were less frequent on the contralateral side of SC-lesioned rats (Mann–Whitney *U*-test, *p* < 0.05) than in control rats. There were no significant differences between whisks on the ipsilateral side of SC-lesioned rats and those of control rats.

The duration of single whisks was also analyzed (Figure 4B). The effects of SC lesions on whisk durations were similar on the ipsilateral and contralateral sides. On both sides, whisks of moderate duration (140–180 ms on the ipsilateral side, 140–200 ms on the contralateral side) were less common (Mann–Whitney *U*-test, *p* < 0.05), and whisks of long durations (260–340 ms on the ipsilateral side, 240–340 ms on the contralateral side) were more common (Mann–Whitney *U*-test, *p* < 0.05), than in the control rats. To rephrase these findings, SC-lesioned rats increased their whisking activities at low frequencies (about 3–4 Hz) and decreased those at moderate frequencies (about 6–7 Hz).

### Coherence of the Right and Left Vibrissal Movements

**FIGURE 5:**
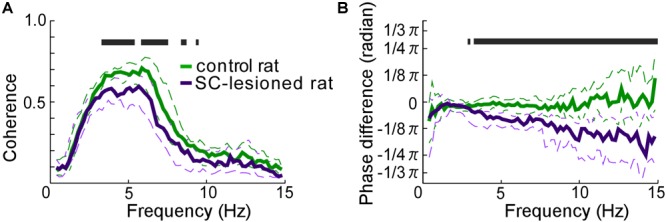
Coordination of vibrissal movements on the right and left sides of the face. **(A)** There was less coherence between the right- and left-side vibrissal movements in SC-lesioned rats (*n* = 16) than in the control rats (*n* = 16). **(B)** Phase differences between right and left vibrissal movements in SC-lesioned rats were statistically larger than those in control rats. Solid lines and dashed lines indicate medians and interquartile ranges, respectively. Black bars show *p* < 0.05 using Mann–Whitney *U*-test. SC, superior colliculus; ipsi, side ipsilateral to the lesion; contra, side contralateral to the lesion.

SC lesions decreased the coherence of vibrissal movements between the right and left sides across a wide range of frequencies (Figure 5A). There were statistically significant reductions in coherence for the middle ranges of frequencies (3.3–5.5 Hz, 5.9–7.6 Hz, 8.4–8.8 Hz, 9.4–9.6 Hz, Mann–Whitney *U*-test, *p* < 0.05). In addition, SC-lesioned rats exhibited differences in kinematic phase between right and left vibrissal movements (Figure 5B). When the rats moved their vibrissae at moderate or high frequencies (>3.3 Hz), the vibrissae on the contralateral side lagged behind the movements of the ipsilateral vibrissae. The delay was about 8.6 ms for movements at a frequency of 5 Hz. The reduction of coherence might be explained by the altered whisking kinematics in the SC-lesioned rats; lower occurrence rate of whisking, increased whisking amplitude and phase delay on the contralateral side.

All of the observed effects of SC lesions are summarized in Table 1, which shows that such lesions mainly affected vibrissal movements on the contralateral side. To explore the neural circuitry underlying these results, we next morphologically analyzed the projection patterns of descending axons that originate from the SC.

**Table 1:** The effects on vibrissal movements of unilateral lesions of the SC are different for the ipsilateral and contralateral sides.

Affected side	Component	Effect
Contralateral	Occurrence rate of whisks	n.s.^a^
	Position	Backward shift of resting vibrissal positions.
	Amplitude	Decrease in moderate-amplitude whisks and increase in large-amplitude whisks.
	Duration	Decrease in whisks of moderate duration and increase in whisks of long durations.
Ipsilateral	Occurrence rate of whisks	n.s.^a^
	Position	n.s.
	Amplitude	n.s.
	Duration	Decrease in whisks of moderate duration and increase in whisks of long durations.

	Coherence	Decrease in coherence between right and left vibrissal movements in SC-lesioned rats.^b^

*n.s., not significant. ^*a*^The number of whisks on the contralateral side is less than that on the ipsilateral side. ^*b*^The phase lag between bilateral vibrissal movements in SC-lesioned rats is larger than that in control rats.*

### Axonal Projections From the SC to the Brainstem

**FIGURE 6:**
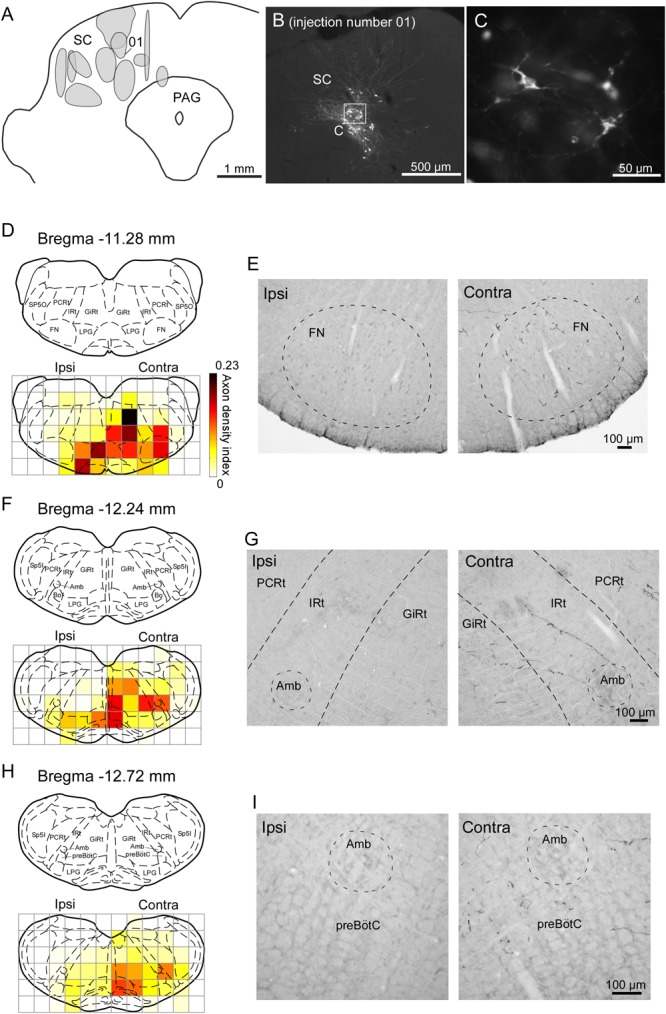
Sindbis virus injections into unilateral SC and the anterogradely labeled axon fibers. **(A)** Light grays on the drawing of the coronal plane indicate the central regions of injection sites. **(B,C)** PalGFP-expressing SC neurons. **(D–I)** Coronal sections at the level of the FN **(D,E)**, IRt **(F,G)**, and preBötC **(H,I)**. Axonal density in each area was expressed as the axon density index (see section “Materials and Methods”). The coronal images at the bottom in panels **D,F,H** were overlaid with the corresponding schematic drawings from the top in those panels (Paxinos and Watson, 2007). Many axonal fibers from the SC were observed in the lower brainstem contralateral to the injection. The two photomicrographs in each row are from the same rat. Amb, ambiguus nucleus; Bo, Bötzinger complex; contra, side contralateral to the lesion; CPGs, central pattern generators; FN, facial nucleus; GiRt, gigantocellular reticular nucleus; ipsi, side ipsilateral to the lesion; IRt, intermediate reticular formation; LPG, lateral paragigantocellular nucleus; PAG, periaqueductal gray; PCRt, parvicellular reticular nucleus; preBötC, pre-Bötzinger complex; SC, superior colliculus; Sp5I, spinal trigeminal nucleus, interpolar part; Sp5O, spinal trigeminal nucleus, oral part.

We injected the recombinant Sindbis virus vector unilaterally into the SC of 11 rats. The locations of all of the injection sites are shown in Figure 6A. The injections were located in the range of 5.8–7.0 mm caudal to the bregma, although the centers of the injections were scattered along the dorso-ventral and medio-lateral directions. We estimated that each injection site contained several tens of infected neurons within the SC (Figures 6B,C). In the present study, we traced labeled axons only in the brainstem caudal to the injection site, because we were primarily interested in the descending circuit from the SC to the FN and the CPGs of whisking. As shown in Figures 6D–I, the SC mostly sent axons to the contralateral side of the brainstem. The descending axons from the SC were mainly distributed in the ventral and medial regions of the lower brainstem, and we confirmed that the labeled axons projected to the FN, IRt, and preBötC. Additionally, many labeled axons reached the parvicellular reticular nucleus, gigantocellular reticular nucleus, and spinal trigeminal interpolaris nucleus on the contralateral side, as well as the lateral paragigantocellular nuclei on both sides. It has been reported that premotor neurons for vibrissal motoneurons are distributed in all of these brain regions (Takatoh et al., 2013).

To compare the SC projection axons to the vibrissal motor related structures in the brainstem with those to oculomotor related structures (Supplementary Figure 1), we calculated axon density index of the PPRF and AN. Axon density index of the areas containing the IRt and FN in the contralateral side were 0.094 and 0.145, respectively, while axon density index of SC projections to the PPRF and AN in the contralateral side were 0.219 and 0.004, respectively. This result suggests that the vibrissal movement related circuit in the brainstem receives considerable projections from the SC, although the density of the projection axons in the PPRF is higher than that in vibrissal motor structures. It should be noted that the SC directly sent many axons to the FN while a motor nucleus of ocular movement, the AN, only receives a few axons from the SC.

**FIGURE 7:**
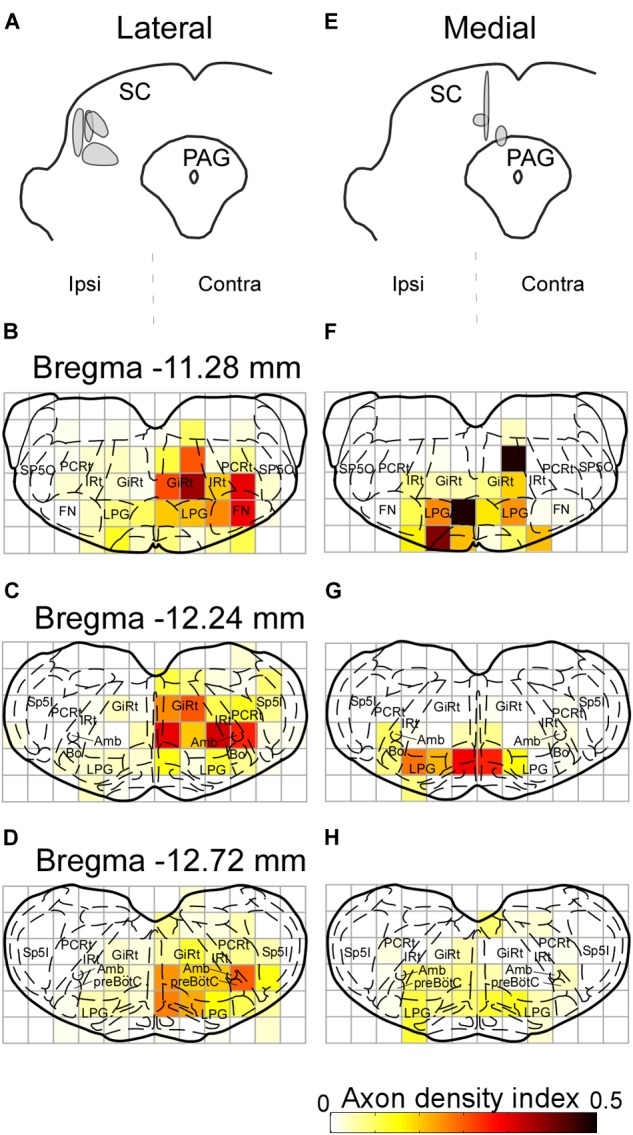
Axonal projections from the lateral and medial parts of the SC. The injection sites of the Sindbis virus into the lateral and medial areas of the SC are shown in panels **A,E**, respectively. Light grays indicate the central regions of the injection. **(B–D)** Lateral SC neurons projected to the contralateral FN, IRt, and preBötC. **(F–H)** Few medial SC neurons projected to the FN, IRt, and preBötC contralateral to the injection. Axonal density in each area was expressed as the axon density index (see section “Materials and Methods”). For the abbreviations, see the legend of Figure 6.

In earlier studies, the SC has been reported to send descending projections not only to the contralateral side but also to the ipsilateral brainstem (Redgrave et al., 1986, 1987; for review see Dean et al., 1989). The contralateral projection is considered to originate in the lateral part of the SC, whereas the cells in the medial part of the SC send descending axons mainly to the ipsilateral side. Accordingly, we extracted cases with injections into the lateral part of the SC (*n* = 4) and those with injections into the medial part (*n* = 3) to compare projection patterns in these two groups. In the former group, labeled axons traversed the midline and descended to the medial regions of the pons and medulla oblongata (Figures 7A–D). We also found that many axon collaterals of labeled neurons spread widely in the contralateral brainstem, which contains whisker-movement related structures, such as the FN, IRt, and preBötC. In contrast, infected neurons in the medial part of the SC sent caudally projecting axons to both sides of the brainstem. Axons from the medial SC were localized principally in the medial part of the brainstem, and only a few axons were observed in the FN, IRt, and preBötC on both sides (Figures 7E–H).

## Discussion

**FIGURE 8:**
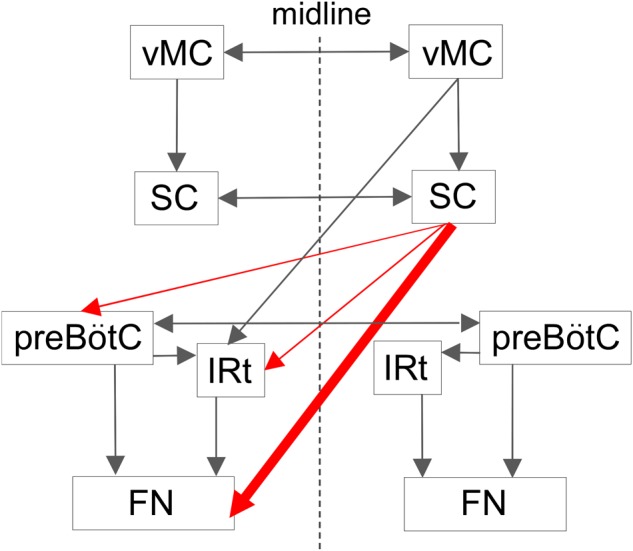
Proposed central projections from the SC for the vibrissal sensorimotor system. The SC receives input from the ipsilateral vMC. The SC and the vMC send their output to the contralateral CPGs and FN, where the vibrissal motoneurons are located. This study indicates that there is a descending circuit from the SC (red lines) that modulates vibrissal movements. Descending pathways from the vMC and the SC are shown on only one side. vMC, vibrissal motor cortex. For the remaining abbreviations, see the legend of Figure 6.

The schematic diagram in Figure 8 illustrates potential circuits for the generation of vibrissal movements. In the present experiment with awake rats, SC lesions mainly affected vibrissal movements on the contralateral side. Our anatomical analysis of descending axons demonstrated that the SC primarily projects to the contralateral brainstem, which contains structures involved in vibrissal movements. Furthermore, we newly evaluated the SC-originated descending axons to the medulla oblongata structures semi-quantitatively, and revealed dominance of the direct projections from the SC to the FN. This architectonic characteristic is in accordance with the results of the vibrissal movement analysis as discussed below.

### Methodological Considerations

We made the lesions in the SC by passing direct electrical current. This method not only lesioned the SC neurons that send descending axons to the lower brainstem, but also damaged other SC neurons. Because the SC projects to the thalamus, anterior pretectal nucleus, zona incerta, and pedunculopontine tegmental nucleus (for review, see Bosman et al., 2011), the present SC lesions may have ablated projection fibers reaching those areas. Given that those structures rarely send direct input to the FN, where the whisking-associated motoneurons are located, we suspect that the destruction of the other projections from the SC made less of an impact than did the destruction of the direct connections to the FN or the descending connections to the whisking CPGs (Moore et al., 2013; Deschênes et al., 2016) from the SC. Therefore, in this report, we discuss the effects of SC lesions on vibrissal movements in relation to the functions of SC-CPGs and SC-FN circuits.

In the present virus-labeling technique, we used a small volume of virus solution for each injection to limit the numbers of infected neurons. Even in these small-volume injections, virus particles spread from the tips of the injection pipettes, and thus the technique resulted in scattered distributions of infected neurons (Figure 6B). Therefore, we could not obtain layer-specific labeling for projection fibers in the SC. The layer-dependent projection patterns have been reported in previous studies, where the descending collicular projections to the lower brainstem and spinal cord were found to derive from the intermediate and deep layers of the SC (Harting et al., 1973; Graham, 1977; Huerta and Harting, 1982; Redgrave et al., 1986). It is reasonable to conclude that, in the present experiment, the labeled axons found in the lower brainstem originated from the intermediate and deep layers of the SC. Overall, the results of the present tract-tracing experiment are in good agreement with previous reports of the projection patterns from the SC (Waldron and Gwyn, 1969; Redgrave et al., 1987; Yasui et al., 1994). Furthermore, our study presents a new analysis of the axonal projection patterns. The virus-labeling technique clearly visualized axons even if only a small volume of virus was injected (and, as such, the number of infected neurons was small). This technique facilitated the quantitative analysis of axonal distributions.

### Relationship Between the Effects of the Unilateral SC Lesion and the Anatomical Basis

The present tract-tracing experiment showed that the descending pathways from the SC mainly reach the contralateral lower brainstem, which contains the whisking-associated areas (Figure 6). This observation may explain the results of the SC lesion study, where most effects of the lesion were exhibited by the contralateral vibrissae. The backward shift of the position of the contralateral vibrissae during resting periods in the SC-lesioned rats (Figure 3) suggests that the baseline activity of the motoneurons that innervate the whisking muscles (intrinsic muscles, Hill et al., 2008; Haidarliu et al., 2010) had decreased. This shift of vibrissal position in the lesion study is considered to relate to the direct input from the SC to the contralateral FN, because the motoneurons for whisking are located in the FN. Whisking behavior is generated by periodic input to the FN from the CPGs of whisking (Moore et al., 2013; Deschênes et al., 2016). The direct connections from the SC to the FN might also affect amplitude control by providing subthreshold input to the FN, which has been suggested to modulate the input/output relation of the neurons (Chance et al., 2002; for review, see Salinias and Sejnowski, 2001). We had suspected that the ablation of direct input from the SC to the FN reduced activity of FN neurons because resting position (angle) of vibrissae, which are protracted by only contraction of intrinsic vibrissal muscle and return to the resting position by elastic nature, was shifted toward the retracted direction in lesioned rats. However, large-amplitude whisks were increased in the contralateral side of the lesioned rats. In oculomotor control, the previous studies in monkeys and cats reported that saccadic amplitudes and neck EMG are affected by the initial eye position (Moschovakis et al., 1998; Corneil et al., 2002; Hadjidimitrakis et al., 2007). Even in vibrissal movement control, it is possible that the resting position affects the size of whisking amplitude. The present increase of whisking amplitude in lesioned rats might be caused by the caudal shift of resting vibrissal position.

The vibrissal movement is also modulated by another higher control center, the vMC, where neurons encode whisking amplitude and/or midpoint (Hill et al., 2011). In the previous study, Hemelt and Keller (2008) showed that microstimulation in the vMC evoked rhythmic vibrissal movements while a sustained vibrissal motion was induced by microstimulation in the SC. Given that the vMC sends descending axons abundantly to the IRt and only slightly to the FN (Hattox et al., 2002; Grinevich et al., 2005), the vMC is considered to take part in vibrissal movement control via the CPGs of whisking in the brainstem. Thus, the mechanism that the SC contributes to motor control of vibrissae is different from that of the vMC.

Whisking CPGs include the preBötC, whose right and left sides are connected by commissural axons, and which control vibrissal movement on both sides with the common basic oscillator (Koizumi et al., 2013; Moore et al., 2013; Deschênes et al., 2016). In the present SC-lesion study, we observed no significant differences in the distributions of whisk durations between the right and left sides, although the whisk durations tended to be longer than those of the normal rats (Figure 4B). Analysis of the coherence of vibrissal movements between the right and left sides revealed that unilateral SC lesions caused desynchronization of the bilateral movements (Figure 5). The decreased coherence in SC-lesioned rats is likely caused by the phase delay on the contralateral side. Assuming that most SC projections to the contralateral brainstem are excitatory, ablation of the SC-brainstem connections may decrease neuronal activity in relevant networks and delay the start of muscle contraction. Phase difference is considered to become larger when rats whisked at a high frequency (Figure 5) because a fixed time difference between right and left side causes a larger phase difference in a high frequency range than a phase difference in a low frequency range.

As for whisk duration, it could become long as whisk amplitude became large in the contralateral side of the lesioned rats. However, it is not clear why elongation of duration was observed also in the ipsilateral side. We speculate that the duration of the ipsilateral side increased following the contralateral side because the commissural connections between the bilateral preBötCs acted to keep the common rhythm in the both side of whisking CPGs.

Given that, in the unilateral SC-lesioned rats, the structures in the medulla oblongata of the contralateral side to the lesion lose input from the SC, one would consider that the lesion causes serious disorders of vibrissal movements in the contralateral side. However, we found no significant differences in number of whisks between the control rats and the contralateral side of lesioned rats, implying a compensation mechanism. Even in the unilateral SC-lesioned rats, the preBötC may keep coordination of the both sides because this structure possesses dense commissural connections, and then contributes to harmonization of activities between the both sides of vibrissal movement circuit in the brainstem (Deschênes et al., 2016). Although undamaged SC mainly sends axons to the ipsilateral side to the lesion, the ablation of input from lesioned SC to the contralateral CPGs might be compensated by the undamaged SC via the commissural connections of the preBötC.

In the ocular movement control, ablation or pharmacological inactivation of the SC induces disorders of eye movements (Albano et al., 1982; Hikosaka and Wurtz, 1985). On the other hand, in the rhythmic vibrissal movements, relatively small changes were caused by the present SC lesion. The most remarkable effect on vibrissal motor control was the caudal shift of resting vibrissal position. This difference might be explained by the differences in SC projection pattern between the oculomotor related structures and vibrissal movement related structures. Motor nucleus for eye movements received only few axons from the SC, while many axons were projected from the SC to the premotor structures (e.g., PPRF). Compared to the oculomotor circuits concerning the SC, vibrissal movement circuits are characterized by direct axonal projections from the SC to the motor nucleus (Figure 8). The SC, in the vibrissal movement control, might mainly act to modulate static parameters (e.g., resting vibrissal position) rather than dynamic parameters (e.g., whisking frequency and amplitude). Future experiments in which activities of each pathway in vibrissal movement circuit are specifically controlled (e.g., by optogenetics) may contribute to identifying the detailed roles of these neural connections.

### The Role of the SC in Vibrissal Movements

In rodents, the SC mediates two types of behavior through sensory input from visual, somatosensory, and auditory areas: *orienting responses*, which direct the sensory apparatus of eyes, vibrissae, head and body to a point of interest, and *defensive responses*, such as avoidance or flight (for review, see Dean et al., 1989). Orienting responses are thought to be generated mainly through the crossed tectospinal pathway from the lateral SC, whereas defensive responses are thought to be mediated through the ipsilateral tectospinal pathway from the medial SC (Dean et al., 1986; Redgrave et al., 1986, 1987; Sahibzada et al., 1986; for review see Dean et al., 1989). The present experiments have revealed that the CPGs and FN received input from cells in the lateral SC on the contralateral side (Figure 7). This suggests that the SC-brainstem circuit for whisking contributes to directing vibrissae toward objects of interest, or to coordinating vibrissal movements with eye, head, and body movements in orienting responses. The SC is a locus where multisensory information are combined and transformed into adaptive motor responses (for review, see Wolf et al., 2015). Although the vMC is also relevant to vibrissal movement control, the functions of the vMC reportedly differ from those of the SC circuits. The vMC functions include motor learning with vibrissae (Huber et al., 2012), initiating vibrissal movements (Sreenivasan et al., 2016), and changing modes of vibrissal motor control (Haiss and Schwarz, 2005). To direct the vibrissae toward sensory stimuli, it is necessary to change the vibrissal positions or whisking patterns between the right and left sides. The results of our lesion study and anatomical analysis suggest that the SC may be involved in vibrissal orientation by making such differences between the right and left.

## Author Contributions

MK performed the experiments. MK and K-iS built the experimental setup. MK and JM analyzed the data. MK and TF designed the study and wrote the paper. AM contributed to the manuscript revisions and editing.

## Conflict of Interest Statement

The authors declare that the research was conducted in the absence of any commercial or financial relationships that could be construed as a potential conflict of interest. The handling Editor declared a shared affiliation, though no other collaboration, with the authors.

## Abbreviations

Amb: ambiguus nucleus
AN: abducens nucleus
Bo: Bötzinger complex
contra: side contralateral to the lesion
CPGs: central pattern generators
FN: facial nucleus
GiRt: gigantocellular reticular nucleus
ipsi: side ipsilateral to the lesion
IRt: intermediate reticular formation
LPG: lateral paragigantocellular nucleus
PAG: periaqueductal gray
PCRt: parvicellular reticular nucleus
PPRF: paramedian pontine reticular formation
preBötC: pre-Bötzinger complex
SC: superior colliculus
Sp5I: spinal trigeminal nucleus, interpolar part
Sp5O: spinal trigeminal nucleus, oral part
vMC: vibrissal motor cortex
