# Supplementary material for: A Descending Circuit Derived From the Superior Colliculus Modulates Vibrissal Movements
Source: Front Neural Circuits. 2018 Nov 22;12:100. doi: 10.3389/fncir.2018.00100 (PMC6262173; doi:10.3389/fncir.2018.00100)
Supplement: Supplementary file 1 [file Data_Sheet_1.docx]

Supplementary Material

A descending circuit derived from the superior colliculus modulates vibrissal movements

Miki Kaneshige, Ken-ichi Shibata, Jun Matsubayashi, Akira Mitani, Takahiro Furuta

*** Correspondence:** Takahiro Furuta: [furuta@dent.osaka-u.ac.jp](mailto:furuta@dent.osaka-u.ac.jp)

# Supplementary Figure

#
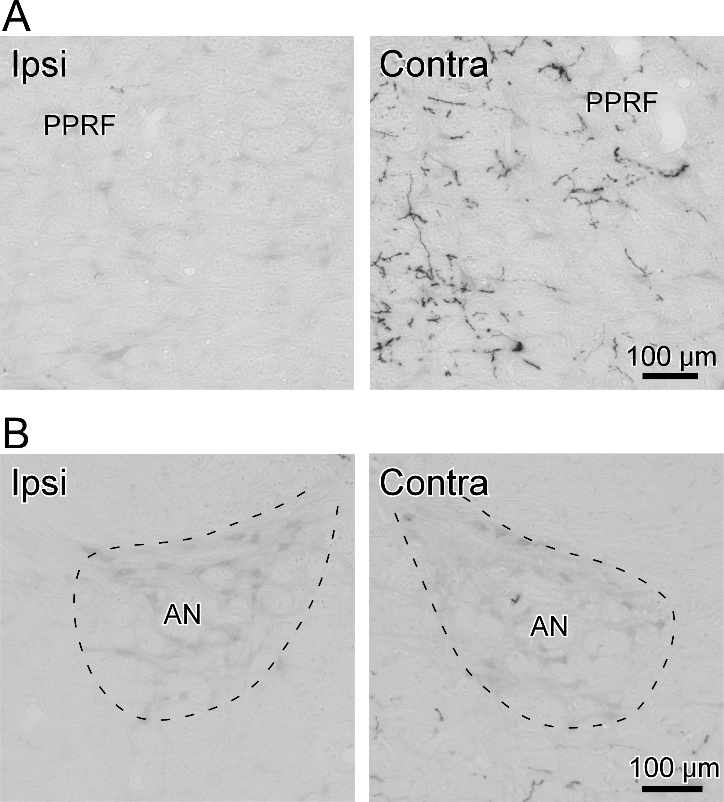


**Supplementary Figure 1. Axon fibers projected from the SC to the oculomotor-associated regions. (A)** Coronal sections at the level of the paramedian pontine reticular formation. Substantial axonal projections were observed in the side contralateral to the injection, whereas sparse axonal projections were distributed in the ipsilateral side. **(B)** Coronal sections at the level of the Abducens nucleus. No and few axon fibers were observed in the side ipsilateral and contralateral to the injection, respectively. The two photomicrographs in each row are from the same rat. Abbreviations: AN, abducens nucleus; contra, side contralateral to the lesion; ipsi, side ipsilateral to the lesion; PPRF, paramedian pontine reticular formation.
